# Hours of Work for Women

**Published:** 1916-03-11

**Authors:** 


### HOURS OF WORK FOR WOMEN.

### Night Work for Nurses and Munition Workers.

The committee which is investigating all sorts of questions connected with the health of munition workers has recently issued two more memoranda (Nos. 4 and 5), which deal respectively with the employment of women and with hours of work. In so far as these memoranda refer to problems exclusively concerned with the output of the munition factories they have but little special interest for institutional workers; but when hours of work in general, and especially hours of women's work, are under discussion, the conclusions reached are bound to touch the institution—as an employer of labour—very closely.

### Night Duty.

The question of night work receives a good deal of attention: on this point the committee assert that night work for women in the factories is undesirable, but for the present is inevitable. The evidence before them is highly conflicting as to the merits of continuous night work as against the system of a fortnightly or monthly change of shift. In favour of continuous night shift is the argument, which has undoubtedly a good deal of force and truth, that after a while the organism accommodates itself to sleeping by day and eating and working by night. On the other hand, it appears that after a month or two on night shift some women get discontented and give up their work unless allowed a turn at day duty. The difficulty is one that has long been familiar to matrons and institutional managers, amongst whom there are just as great differences of opinion on the subject as among factory managers. The writer's belief is that two months at a time is quite long enough for any hospital nurse to remain on night duty, and that in no circumstances should three months at a stretch ever be exceeded, except in the case of nurses whose training is completed and who expressly volunteer for long spells of night work. It is of interest to note that the committee have had in mind the night work of hospital nurses; for they state that comparisons cannot fairly be drawn between industrial night work and the night duty of nurses. "Not only," says the memorandum, "are nurses a selected and trained body of women, but the disciplined conditions of their lives are not those either of the factory or the working-class home." If we follow the drift of this remark correctly, it means that nurses may be legitimately subjected to greater strain than munition workers, because their liberty has been much more completely surrendered and because the fittest only of them have survived! Incidentally, the majority of those on night duty in hospitals are not "trained," but are in course of being trained. The recommendation of the committee on this topic of alternation between day and night shifts is non-committal; they consider that the matter is one which must be largely dealt with locally on social considerations.

On the subject of the hours of women's labour, the committee are definite enough, however. Long hours, particularly when worked during the night, are perhaps the chief factors in fatigue; and the committee hold that in the interest of the output and of the work alike they should be restricted within proper limits, and that there should be adequate cessation from work at each week-end in addition to periodic holidays. Of the various systems of organising women's work, it is stated that the eight-hour shift yields the best results in the long run. The strain of night work is sensibly diminished, greater vigour of work is maintained throughout the shift, and there is less liability to accident. This emphatic statement of opinion applies primarily, of course, to munition workers; but it is equally true of nurses, and those institutions which have already acted on the principle have not only earned the gratitude of their nursing staffs, but have also reaped the benefit of a higher standard of nursing efficiency.

### Administrative Details.

There are many other points dealt with in the memoranda which recall to anyone familiar with hospital administration similar or cognate questions of nursing organisation. The committee have something to say, for instance, on the subject of the lifting of weights by women, and on the effects of prolonged standing. They go rather deeper into the provision of suitable meals and of sufficient intervals in which to eat them. They touch here on what is at too many hospitals a very weak spot in the administration, though conditions are, we believe, gradually tending towards improvement in this as in various other respects. All through the committee's memoranda there are evidences of a thoroughly conscientious effort to raise the conditions of labour (male and female), to promote efficiency and to eliminate wastage, and to conserve the health of the worker, because it is one of the most precious of the assets of the State. No one would for a moment decry the value and the necessity of such an effort; but to those who know the severity of the hospital nurse's training, the way in which she surrenders her liberty for a term of years while training, and the pittance which too often she receives in return, it does seem as though the munition worker is a favoured, almost a pampered individual. The war is, of course, responsible for the attention bestowed upon the munition worker; but the nurse is every whit as indispensable to its prosecution. Let it be hoped that one of the lessons it will teach us may result in better conditions and hours of labour for the latter as well as for the former. None know better than readers of The Hospital the splendid services and sacrifices of the nursing profession, and the wide differences between different training schools, some of which have much to learn from the memoranda of the Health of Munition Workers Committee.

